# Low-dose Naltrexone: An Alternative Treatment for Erythrodermic Psoriasis

**DOI:** 10.7759/cureus.3943

**Published:** 2019-01-23

**Authors:** Eduardo P Beltran Monasterio

**Affiliations:** 1 Dermatology, Universidade Gama Filho (instituto De Pesquisa E Ensino Médico), Florianopolis, BRA

**Keywords:** ldn as a new treatment option for psoriasis, erythomatous psoriasis and ldn, ldn, ldn in dermatology, ldn for erythrodermic psoriasis

## Abstract

This clinical case demonstrates the benefits of patient treatment with low-dose naltrexone (LDN) used in erythrodermic psoriasis. A patient with a confirmed history of psoriasis by histopathology was treated with 4.5 mg of LDN during six months follow-up after an erythrodermic psoriasis flare-up. The patient showed significant improvement in her flare-up and psoriasis remission after only three months of 4.5 mg of LDN on a daily basis. Low-dose naltrexone (LDN) has proven to be a great ally in treating erythrodermic psoriasis flare-ups as an alternative treatment with less collateral side effects.

## Introduction

Psoriasis is a chronic, inflammatory, autoimmune skin disease that results in the of formation of white-silvery, dry, itchy patches. Approximately, 2%-3% of the population develops the disease according to the National Psoriasis Foundation (NPF) [1]. The mechanisms involved in the pathogenesis of this condition is still not well-understood, but it is known to be a polygenic disorder with triggering environmental factors such as trauma, infections, medications, and emotional stress.

Several clinical expressions exist, and the characteristic lesion found in patients with psoriasis is the presence of erythematous-scaly (silvery-white) patches found on the skin. These lesions may usually have a chronic form of manifestation and rarely evolve to an acute form known as erythrodermic psoriasis. It generally appears on people who have unstable plaque psoriasis, i.e., in only 3% percent of the people who have psoriasis. Individuals having an erythrodermic psoriasis flare-up should see a doctor immediately. This form of psoriasis can be life-threatening according to the National Psoriasis Foundation (NPF) [2].

Erythrodermic psoriasis is a condition involving practically the entire skin body surface, which can sometimes lead to constitutional symptoms. It is known to a be a severe and dangerous form of psoriasis, which may be life-threatening. In this particular clinical case, we shall discuss the experience of using manipulated oral low-dose naltrexone (LDN) in combined treatment with topical colloidal oat cream and oral hydroxyzine in a patient with severe erythrodermic psoriasis.

Ogawa et al. [3] have described psoriasis histologically by the presence of dendritic and T-cells with increased intralesional levels of T-helper cells, interleukins, and tumor necrosis factor (TNF) at the site of the skin plaque. These immunological markers serve as therapeutical targets for psoriasis treatment. Methotrexate is one of the first and low-cost end drugs that has been used for certain forms of psoriasis, but then again has shown to have severe adverse side effects. Alanna et al. [4] have shown that newer drug therapies have been developed for the treatment of psoriasis through immune-modulating biologics such as adalimumab, ustekinumab, and secukinumab. Despite the high cost of these newer drugs and promising results, not all patients can benefit from them or can tolerate these medications. This has led some physicians to try unconventional anti-inflammatory drugs, such as low-dose naltrexone (LDN), in the treatment of autoimmune diseases, including psoriasis. Significant and credible evidence and research published on Low Dose Naltrexone Research Trust [5] has shown repeatedly the international medical community the promising benefits of this drug.

## Case presentation

The following clinical case is of a 38-year-old, white female patient from the municipality of São Pedro de Alcântara, State of Santa Catarina, Brazil (South America). She worked as a part-time car mechanic and as a security guard. The patient had a long history of psoriasis since the age of 22, which was later confirmed by histopathology. She consulted many dermatologists previously, having used a large variety of topical steroidal creams that showed little benefits.

During her last visit to her dermatologist, she was put on oral methotrexate 7.5 mg/week as an initial dose in association with 5 mg folic acid/day with clobetasol 0.05% cream twice a day. The patient received medical treatment through the public health care system (Sistema Único de Saude - SUS) located at Santa Teresa Dermatologic Hospital in São Pedro de Alcântara, Santa Catarina, Brazil.

The patient mentioned that she showed poor results after three months of treatment. The patient felt that it was necessary to interrupt the medication by her physician due to the side effects of methotrexate (anemia, thrombocytopenia, and leukopenia). After two weeks of taking a suspension of methotrexate, the patient showed acute worsening of her condition and went to the Family Medicine Health Care Clinic (Maria Rasveiler Junckes) where she consulted another dermatologist on June 22, 2016.

**Figure 1:**
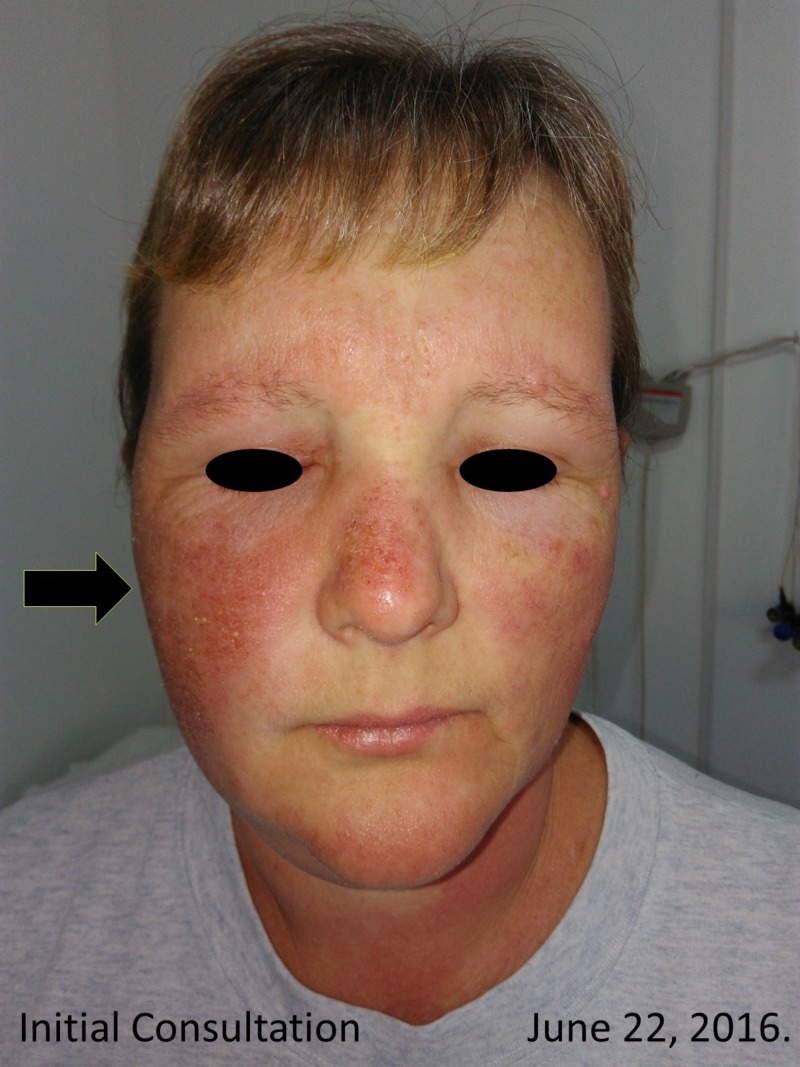
Initial consultation facial photo This picture was taken on initial consultation. Dry skin with inflammatory facial edema and superficial scaling was evident, as shown by the arrow. The patient had recently suspended methotrexate treatment.

**Figure 2:**
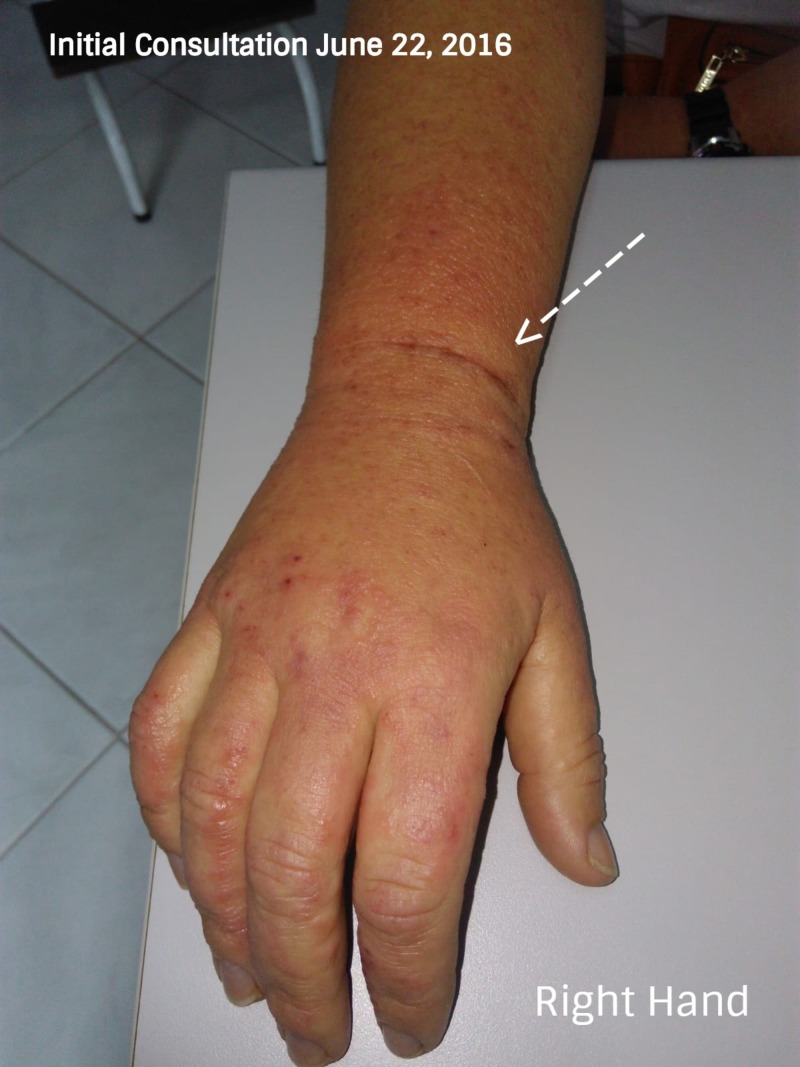
Bilateral edema of upper and lower extremities (Initial consultation) Bilateral edema of the upper and lower extremities was evident, associated with itching and redness, as shown by the arrow. The patient mentioned having some difficulty in making a fist with her hand.

**Figure 3:**
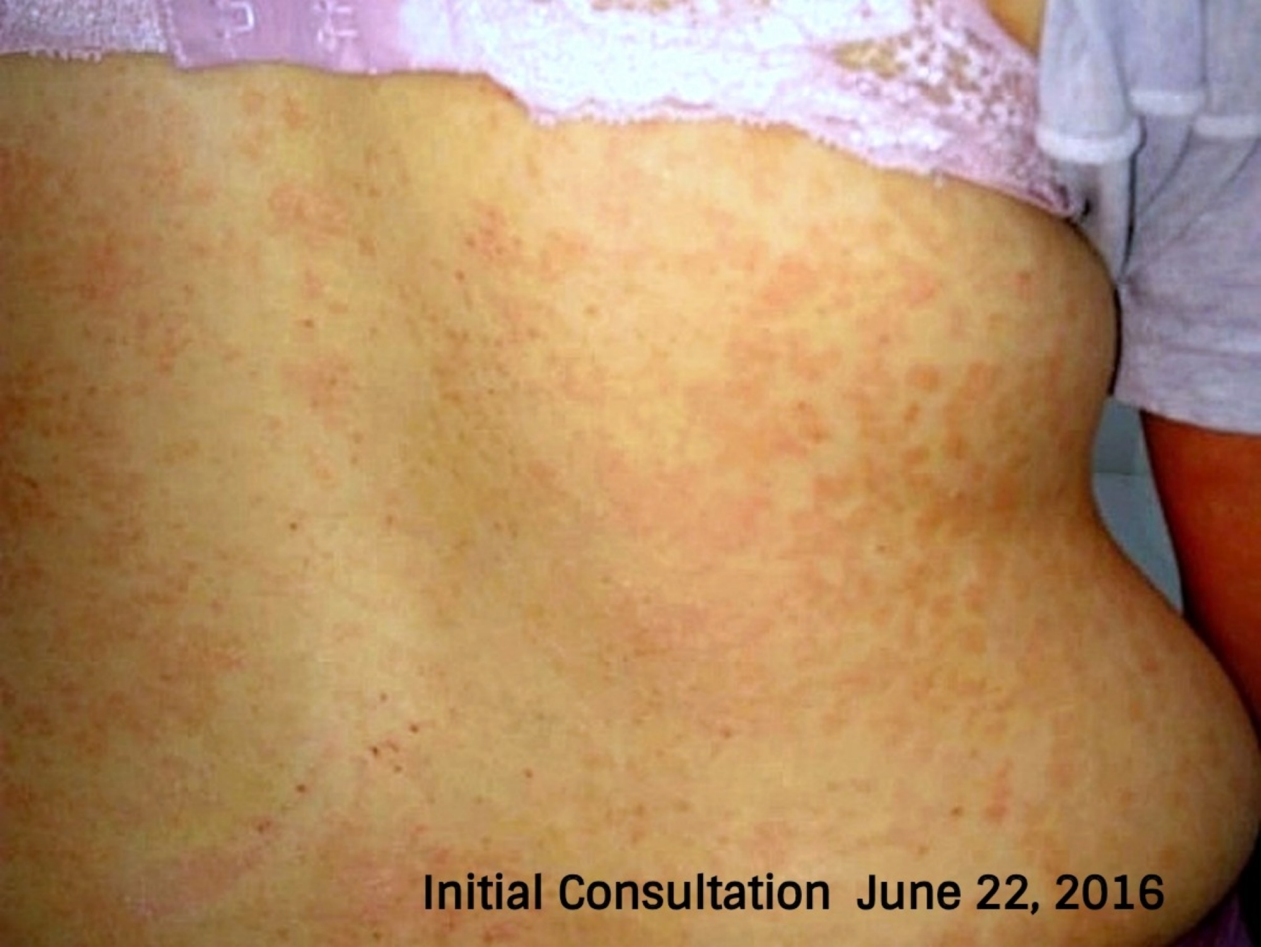
Initial consultation photo of the dorsal back In this photo, we can appreciate the presence of disseminated erythematous plaques throughout the patient's entire body.

On initial physical examination, the patient showed signs of facial swelling (Figure 1), predominantly on the right side of her face with significant "wet" edema of the upper and lower extremities (Figure 2). The patient referred to having "shivers" and severe itching and joint pain with disseminated lesions notated throughout almost all her body surface. Her Psoriasis Area Severity Index (PASI) score was of 48 (Figure 3).

Erythrodermic psoriasis is a condition that sometimes may "throw off" the body's chemistry, causing proteinuria with fluid loss and leading to severe illness. Infection is an important aspect to be taken into consideration, which can bring upon pneumonia and congestive heart failure. 

The patient was monitored closely at our clinic and regular laboratory evaluations were performed to observe any significant metabolic changes. The patient was very concerned about her condition and expressed this verbally, refusing to take any immunosuppressive cytotoxic drugs, such as methotrexate, which was prescribed previously. At this moment, a low-dose naltrexone (LDN) regimen was the only "open-door" option for her and her family, due to the less aggressive side effects of methotrexate.

Treatment

Oral low-dose naltrexone (LDN) was prescribed at a starting dose of 4.5 mg every night with 25 mg of hydroxyzine every 12 hours, with topical colloidal oat cream every eight hours. Her vitals signs kept within normal ranges at all times and showed progressive normalization of her laboratory values. Initial renal function showed mild proteinuria with elevated serum urea and creatinine that normalized throughout the following two weeks. Her folate levels were very low as well. Congestive heart failure and infections were excluded by electrocardiogram (ECG), chest X-ray, and laboratory results. A daily follow-up was done during the first 10 days, followed by a 20-day, three-month, and, finally, six-month follow-up.

Results

After 10 Days of Treatment

The patient showed significant improvement after 10 days of 4.5 mg of LDN orally every night. She no longer mentioned an itchy sensation and discontinued hydroxyzine at nine days of treatment. Signs of regression of the facial swelling and edema of upper and lower extremities were notable after 10 days. Like in the Metze et al. study [6], LDN has been proven to eliminate these symptoms.

After 20 Days of Treatment

**Figure 4:**
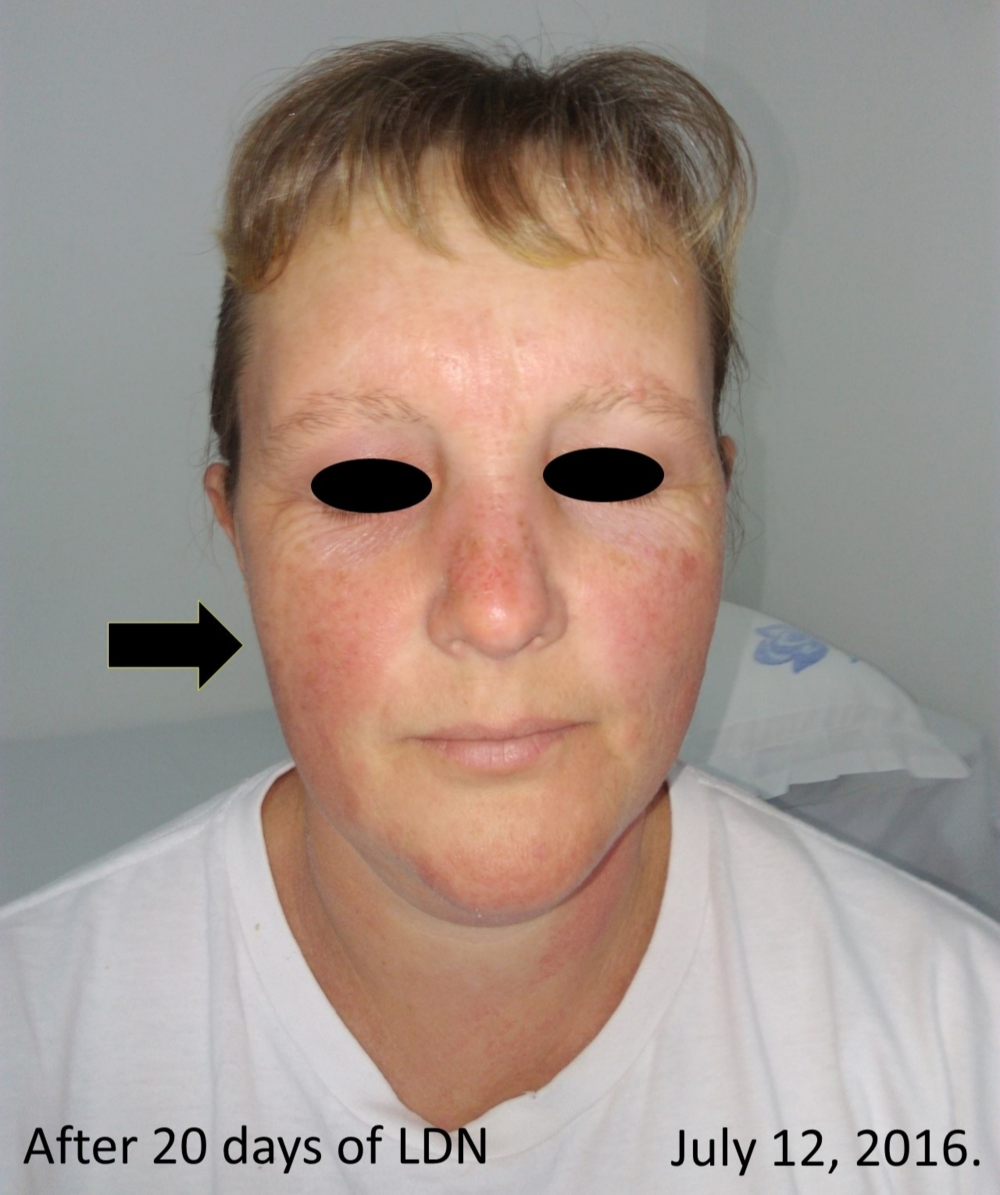
After 20 days treatment with 4.5 mg LDN orally. In this picture, we can observe how low-dose naltrexone (LDN) has shown noticeable improvement in her facial edema. Facial redness had improved by more than 50%, as shown by the arrow. The patient was symptom-free of itching.

**Figure 5:**
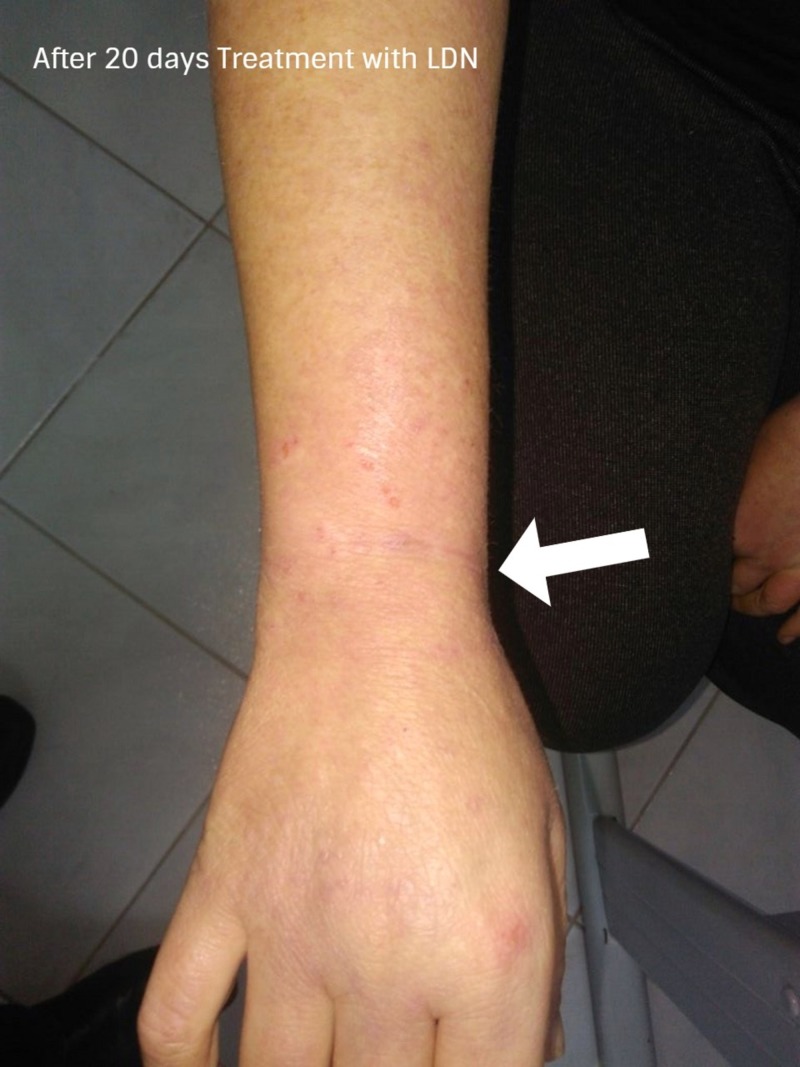
After 20 days of treatment with LDN Significant improvement of bilateral upper extremity edema with an important reduction in the itching sensation obtained with low-dose naltrexone (LDN).

**Figure 6:**
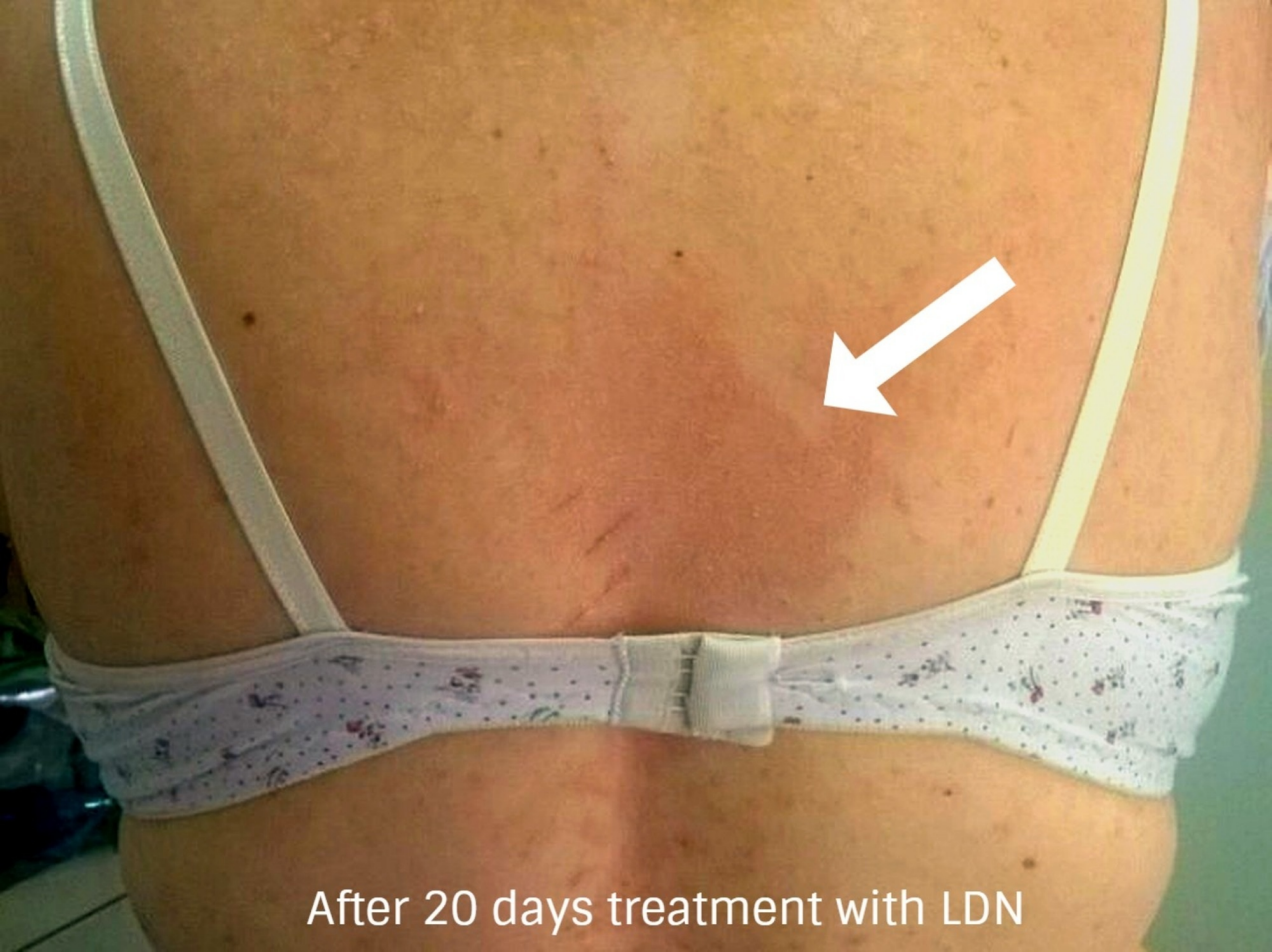
After 20 days of treatment with low-dose naltrexone (posterior dorsal back region) In this photo, we can easily identify an important reduction after 20 days of low-dose naltrexone (LDN) in disseminated erythematous plaque formation. Only a solitary main plaque was found in the central dorsal posterior region of the back as denoted by the arrow.

The patient showed complete improvement after 20 days of treatment with 4.5 mg of LDN orally every night and applying topical colloidal oat cream on a regular, daily basis. The Phan et al. study [7] on antipruritic treatment with systemic mu-opioid receptor antagonists, such as LDN, has proven its benefits as an antipruritic agent. Noticeable reduction in facial edema (Figure 4) by more than 50%, upper extremity swelling (Figure 5), and considerable reduction of disseminated pruritic plaques on the skin surface (Figure 6) were also evident.

After Three Months and Six Months Treatment

**Figure 7:**
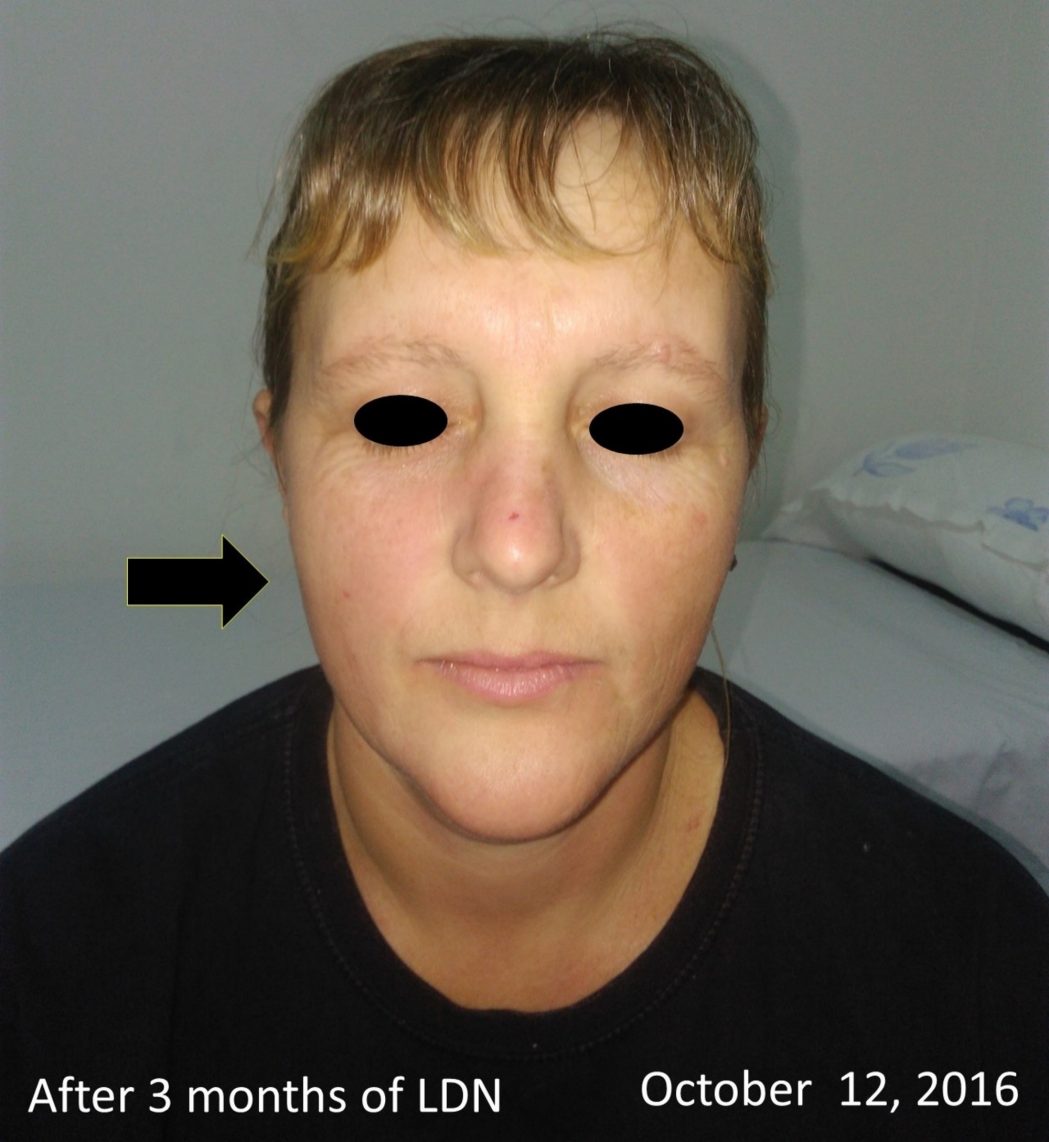
Picture after three months of treatment with 4.5 mg LDN orally In this picture, we can see complete remission of the disease, as pointed to by the arrow after three months of low-dose naltrexone (LDN). Symptom-free (Psoriasis Area Severity Index (PASI) score = 0). The patient was compliant with treatment and did not refer to any side effects of LDN during her treatment. The patient was very pleased with her results.

**Figure 8:**
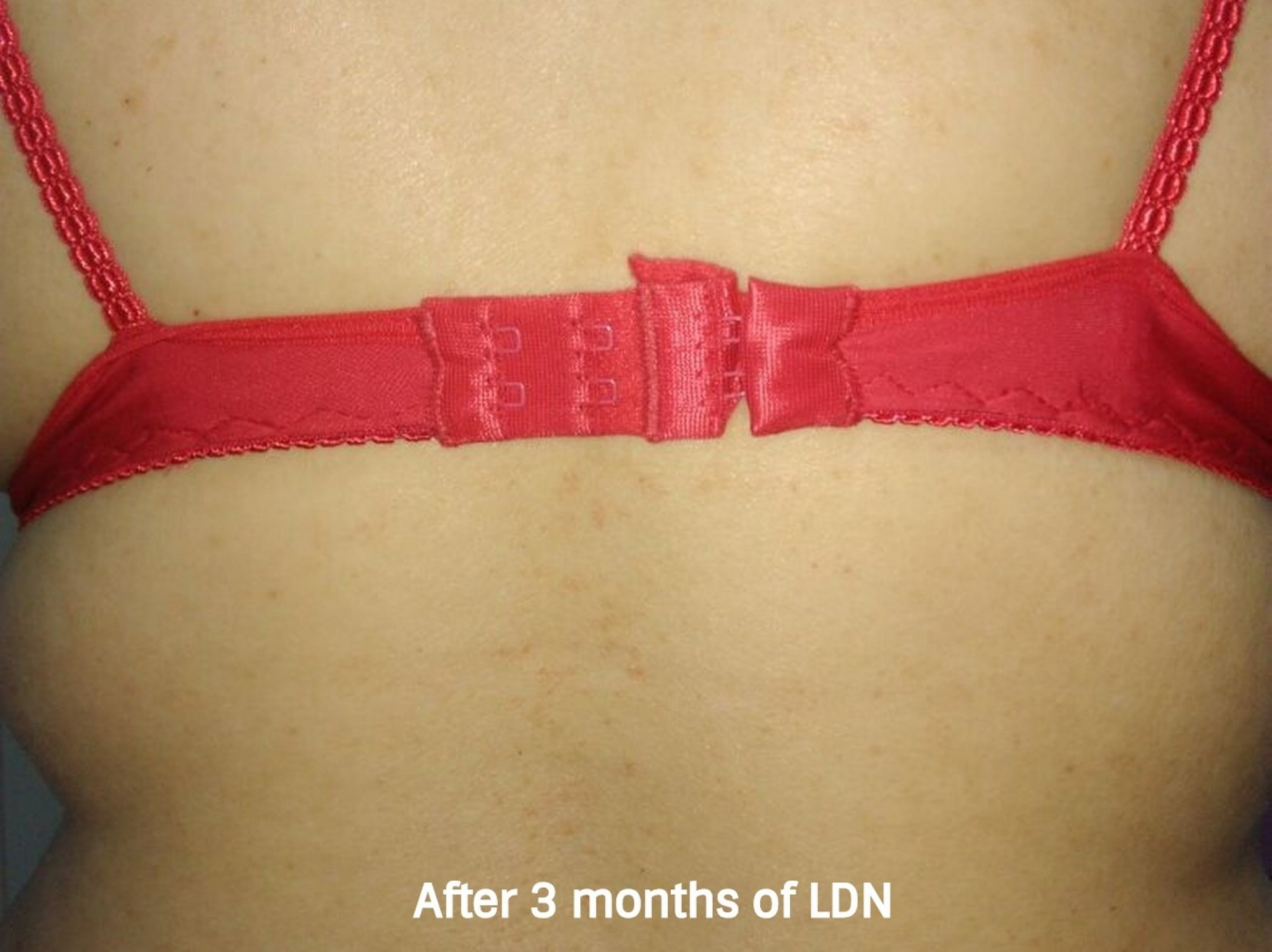
After three months of treatment with low dose naltrexone In this photo, we can notice complete remission of the disease after three months of low-dose naltrexone (LDN) treatment. The patient was symptom-free.

A follow-up was done at three months and six months consecutively after an acute "flare-up" episode. During the consultation, she showed complete psoriasis remission at three months (Figure 7), with a Psoriasis Area Severity Index (PASI) score of 0 (Figure 8). 

**Figure 9:**
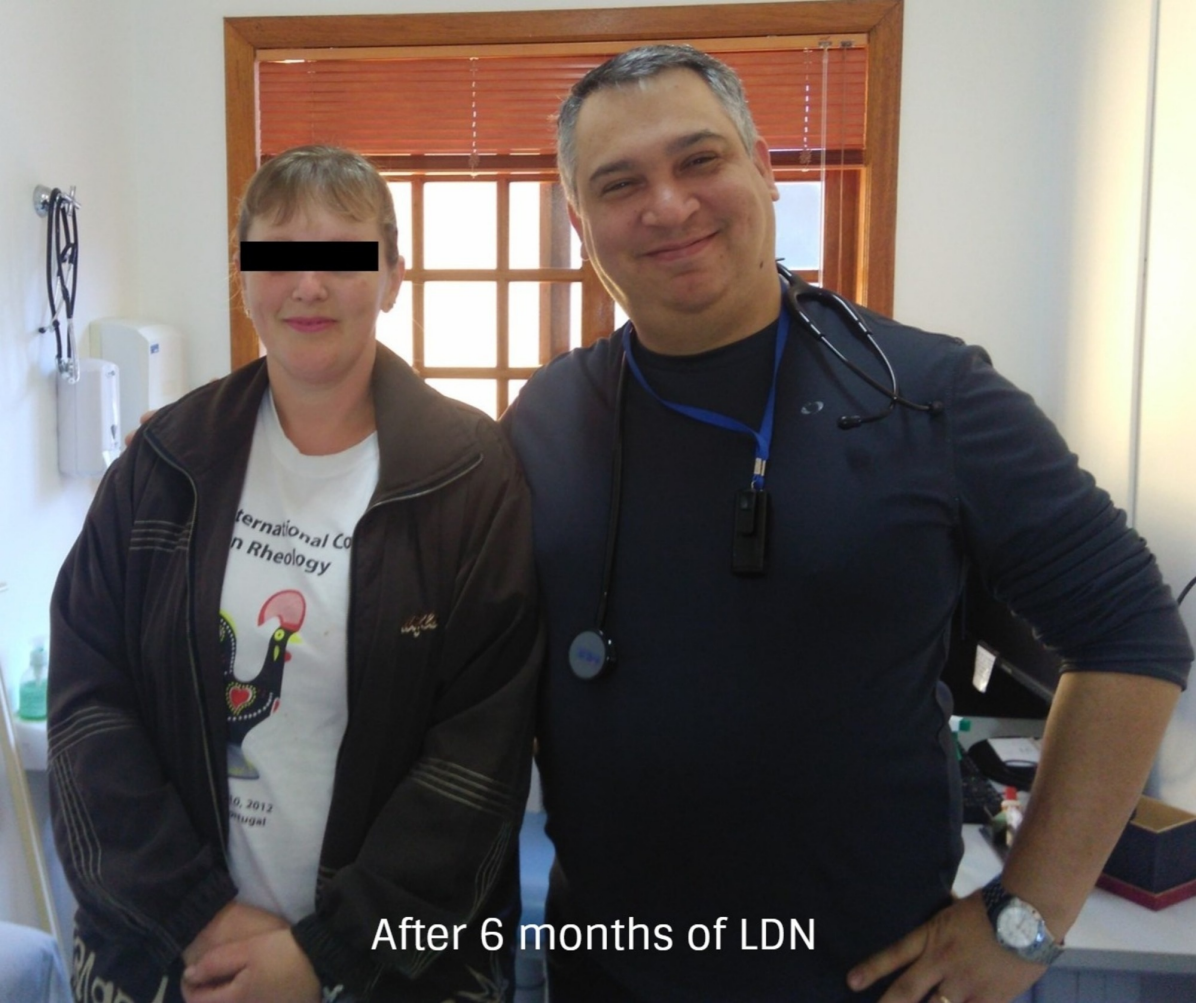
After six months of treatment with 4.5 mg LDN orally After six months of follow-up of low-dose naltrexone (LDN) treatment, the patient showed no signs of psoriasis. Her Psoriasis Area Severity Index (PASI) was 0. Complete remission of the disease was evident. The patient expressed much content with her results and all lab work came back normal.

The same clinical condition was evident after six months of LDN treatment (Figure 9). No side effects of LDN were referred to or observed throughout the whole treatment. Quality of life was improved dramatically by having a profound positive impact on her daily routine. The patient returned to work after 20 days of treatment. It is important to note that the patient used to work as a security corrections officer at night and worked as a car mechanic during the day. Having left her night job also played an important role in the recovery of her condition.

## Discussion

The initial, acute flare-up (trigger) of this clinical case probably was due to the side effects and abrupt withdrawal of conventional treatment with methotrexate. Laboratory findings showed drug-induced pancytopenia (anemia, thrombocytopenia, and leukopenia). Renal function showed proteinuria with mild serum elevation of urea and creatinine values. Erythrocyte sedimentation rate (ESR) was normal. Serum uric acid was slightly elevated. ECG showed a normal sinus rhythm with normal cardiac enzymes. The histopathology of the skin showed hyperkeratosis, parakeratosis, and acanthosis, with an intense presence of neutrophils in the epidermis. The thinning of the epidermis overlying the dermal papillae with vessels close to the epidermis and elongated rete-ridges mitotic was seen on her biopsy.

The diagnosis of erythrodermic psoriasis is mainly clinical. The clinical features that were observed in this case showed the presence of generalized, disseminated erythematous plaques throughout the patients' entire body. The clinical findings of facial edema along with the presence of small vesicular eruptions of the upper and lower extremities were suggestive of fluid retention. The severe disseminated redness throughout the body was contained due to the prompt use of LDN. The patient also mentioned a severe itching sensation, joint pain along with cold "shivers" on the initial consultation, suggestive of the body trying to maintain a normal temperature.

Another important aspect noted is that stressful working conditions can easily be a potential trigger in the resurfacing of any autoimmune disease such as psoriasis. It is very important that the attending physician addresses, evaluates, and identifies any precipitating factors that may induce or cause emotional stress in the patient. Adequate control of the emotional component and a balanced healthy lifestyle is crucial in avoiding future flare-ups. LDN has proven to help maintain the patient´s psoriasis in remission, having a profound positive impact on her daily activities and quality of life.

As mentioned by the Younger et al. [8], the following three theoretical components are of the most relevance regarding the LDN mechanism of action: 1) action on opioid receptors to increase the release of β-endorphins; 2) ability to reduce pro-inflammatory cytokines and increase anti-inflammatory cytokines; and 3) regulation of the opioid growth factor (OGF)/opioid growth factor receptors (OGFr) axis. The increase of β-endorphins for joint pain management is of significant therapeutical importance, having a big impact on the quality of life of this patient. The role that LDN plays in downregulating the multiple pro-inflammatory cytokines is a fundamental aspect in the attempt to balance out and normalize the cutaneous inflammatory state in erythrodermic psoriasis. LDN has shown us that the opioid growth factor-opioid growth factor receptor (OGF-OGFr) axis is a key element that plays a very important role in maintaining a delicate balance in the upregulation and downregulation of the immune system. Little attention has been paid to the development of newer drugs that can act upon this axis.

Having obtained these results throughout the six months of treatment with LDN should be an eye opener for future LDN-prescribing physicians. LDN has proven to have strong potential therapeutical benefits and should encourage more physicians to consider the use of LDN as alternative therapy for patients who cannot tolerate conventional mainstream medicine. 

## Conclusions

This clinical case study has clearly shown the benefits of 4.5 mg low-dose naltrexone for erythrodermic psoriasis. A remarkable improvement was documented by the twentieth day of treatment. Complete remission of the disease was evident by three months, with no signs of plaques anywhere on the patient's body. Her PASI score improved rapidly, with a score of 0 along with her normal laboratory results, by the third month. No secondary side effects were reported at any time during treatment.
